# Conversational ontology operator: patient-centric vaccine dialogue management engine for spoken conversational agents

**DOI:** 10.1186/s12911-020-01267-y

**Published:** 2020-12-14

**Authors:** Muhammad Amith, Rebecca Z. Lin, Licong Cui, Dennis Wang, Anna Zhu, Grace Xiong, Hua Xu, Kirk Roberts, Cui Tao

**Affiliations:** 1 The University of Texas Health Science Center at Houston, School of Biomedical Informatics, 7000 Fannin Suite 600, Houston, 77030 TX USA; 2 Washington University School of Medicine, 660 S Euclid Ave, St. Louis, 63110 MO USA; 3 Texas Tech University Health Sciences Center El Paso, 4801 Alberta Ave 3rd Fl, El Paso, 79905 TX USA; 4 Southern Methodist University, 6425 Boaz Lane, Dallas, 75205 TX USA; 5 University of Texas, 110 Inner Campus Drive, Austin, 78705 TX USA

**Keywords:** Ontology, Patient provider communication, Dialogue management, Natural language processing, Semantic web, Question-answering, Software agents, Human computer interaction, Vaccines

## Abstract

**Background:**

Previously, we introduced our Patient Health Information Dialogue Ontology (PHIDO) that manages the dialogue and contextual information of the session between an agent and a health consumer. In this study, we take the next step and introduce the Conversational Ontology Operator (COO), the software engine harnessing PHIDO. We also developed a question-answering subsystem called Frankenstein Ontology Question-Answering for User-centric Systems (FOQUS) to support the dialogue interaction.

**Methods:**

We tested both the dialogue engine and the question-answering system using application-based competency questions and questions furnished from our previous Wizard of OZ simulation trials.

**Results:**

Our results revealed that the dialogue engine is able to perform the core tasks of communicating health information and conversational flow. Inter-rater agreement and accuracy scores among four reviewers indicated perceived, acceptable responses to the questions asked by participants from the simulation studies, yet the composition of the responses was deemed mediocre by our evaluators.

**Conclusions:**

Overall, we present some preliminary evidence of a functioning ontology-based system to manage dialogue and consumer questions. Future plans for this work will involve deploying this system in a speech-enabled agent to assess its usage with potential health consumer users.

## Background

In normal human interaction, speech is a natural modality for us to communicate to each other. According to research, more information can be communicated in less time than printed material [1–3]. Face-to-face communication between a health providers and patients is an important factor in improving health outcomes. This type of communication is helpful in personal interaction between the patient and provider when discussing the human papillomavirus (HPV) vaccine which mitigates cancers caused by the HPV virus in adulthood, and it has been reported to encourage vaccine uptake [4]. Also, provider communication is recommended by the President’s Cancer Council to increase vaccination uptake [5]. Despite the recommendations and benefits of the HPV vaccine, the vaccination rates are below the 80% coverage rate promoted by the Healthy People 2020 report [6]. This is complicated with the limited time that health care providers have to discuss the HPV vaccine with health consumers, with just a third of the patients receiving a discussion about the HPV vaccine during their visit [4]. One experimental solution is our proposition for a speech-enabled dialogue system embodied in a software agent that could facilitate the communication task of counseling on the HPV vaccine during the patients’ clinical visit.

Spoken dialogue system is defined as “a system [that] enables a human user to access information and services that are available on a computer or over the Internet using spoken language as the medium of interaction” [7]. Earlier, we developed an ontology for dialogue, called the Patient Health Information Dialogue Ontology (PHIDO), that can potentially handle dialogue flow and contextual dialogue information for a software agent. PHIDO is an application ontology based on our previous simulation study with a drone-operated conversational agent [8, 9] (Wizard of OZ experiment^1^[10]). PHIDO provides the basic building blocks to create a framework of dialogue interaction for a user conversing with a machine. We used PHIDO to create a reusable model of our HPV vaccine counseling. This model contains three basic speech tasks that can be linked together to form a discussion. Later, this ontology can be integrated with health intervention models to build upon and validate these models.

**Fig. 1:**
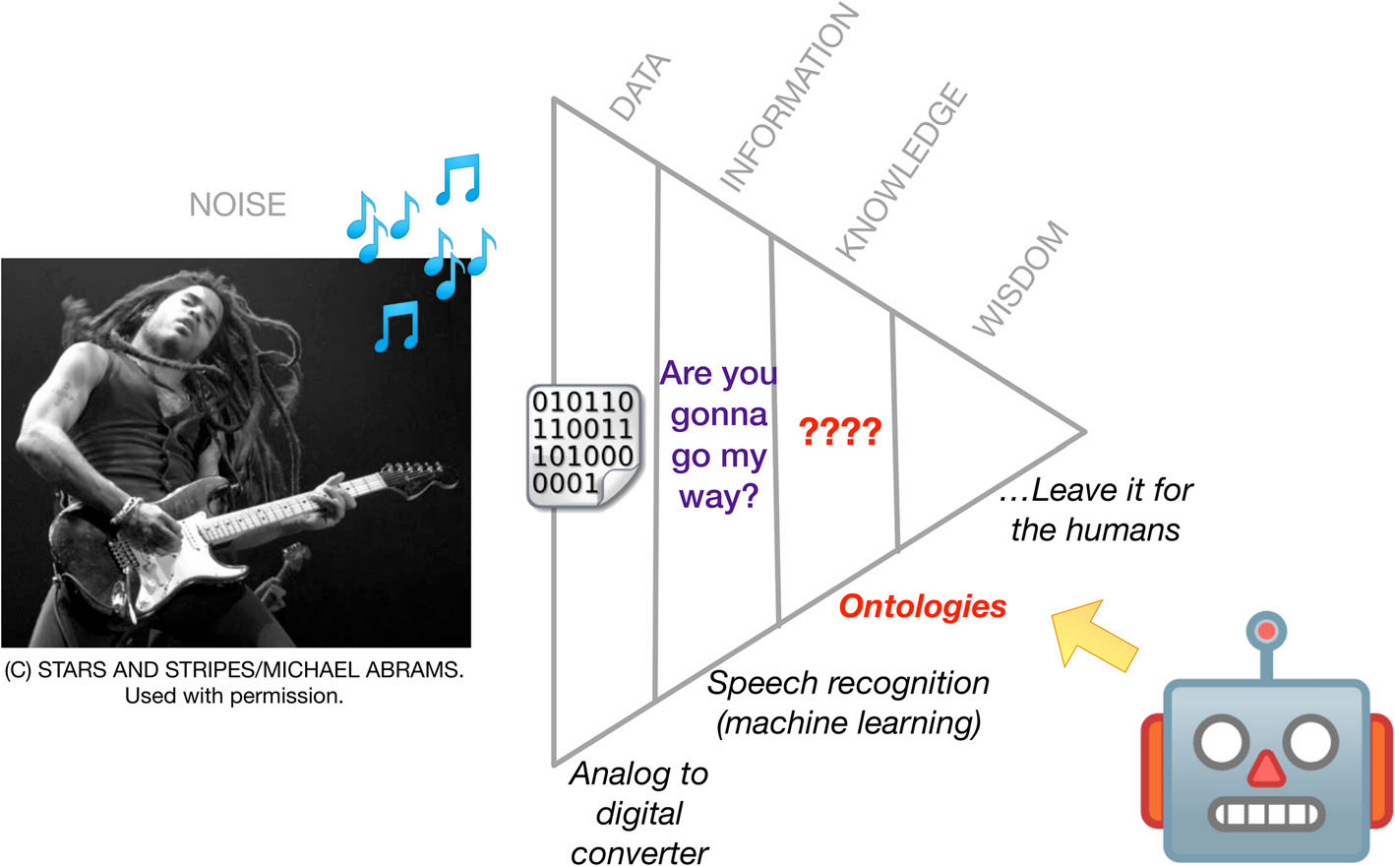
Application of the knowledge pyramid for agents. Concert photograph (“Lenny Kravitz at the Festhalle in Frankfurt Germany, March 20, 1996”) [13] by Michael Abrams is under copyright by Stars and Stripes/Micheal Abrams, and written permission was granted to use and adapt the photograph

Ontologies in the biomedical field have primarily supported efforts related to text-mining and data analytics. However, ontologies have also been known to provide support in automated planning – an AI sub-field for using model-based behavior methods for agents. For example, Olivares-Alarcos and colleagues recently reviewed and identified ontologies for mechatronic-related research [11]. Essentially, ontologies can provide software agents with intelligence and reasoning on how to respond in an environment with other virtual or physical agents, along with sharing an understanding of the environment among the agents. Figure 1 elaborates on this notion with the classic knowledge pyramid in an agent-based context (Fig. 1), where we show how ontologies inhabits a unique role for software agents in the evolution of information on the knowledge pyramid [12]. In the example, an artist playing music emits audio noise (*Noise*) that can be translated into digital format by a robot’s analog-to-digital converter (*Data*). The digital data can be further processed by the machine’s speech recognition software and converted into string text (*Information*). However, the machine needs to know the rules on how to react and behave when presented with information (*Knowledge*).

In this paper, we have developed a prototype software engine that utilizes the PHIDO model to coordinate conversational behavior. The software engine aims to be a plan-based, deterministic system that will initiate and direct the dialogue with the user. This engine is a prototype that we plan to integrate into a device to provide it with the intelligence to discuss health information with a patient autonomously. This engine will not only coordinate the dialogue exchanges with the user, but also answer various vaccine questions from the user. This task is facilitated by a question-answering (QA) subsystem for ontologies. We will use our previously developed ontology VISO-HPV (Vaccine Information Statement Ontology for HPV) [14] as a knowledge base for the question-answering subsystem. VISO-HPV is built upon the TBox-level of Vaccine Information Statement Ontology (VISO) [15] which is a knowledge base of patient-level vaccine knowledge sourced from Vaccine Information Statements (VIS).

We propose the following questions:

- *Could an ontology-based dialogue engine provide essential functions for HPV vaccine counseling – communicate health information to the user, answer questions, and transition to another health topic?*
- *Could the engine’s question-answering subsystem provide satisfactory responses for most of the consumer questions?*

## Methods

In our prior work [16], we described the various utterance and speech task classes and their object and data property links to coordinate the dialogue. In addition, we described a transition mechanism that utilizes the PHIDO to enact a conversation with the user. This transition mechanism is now implemented in the Conversational Ontology Operator (COO), a software engine that manages the dialogue interaction for the agent.

### Dialogue interaction method

**Fig. 2:**
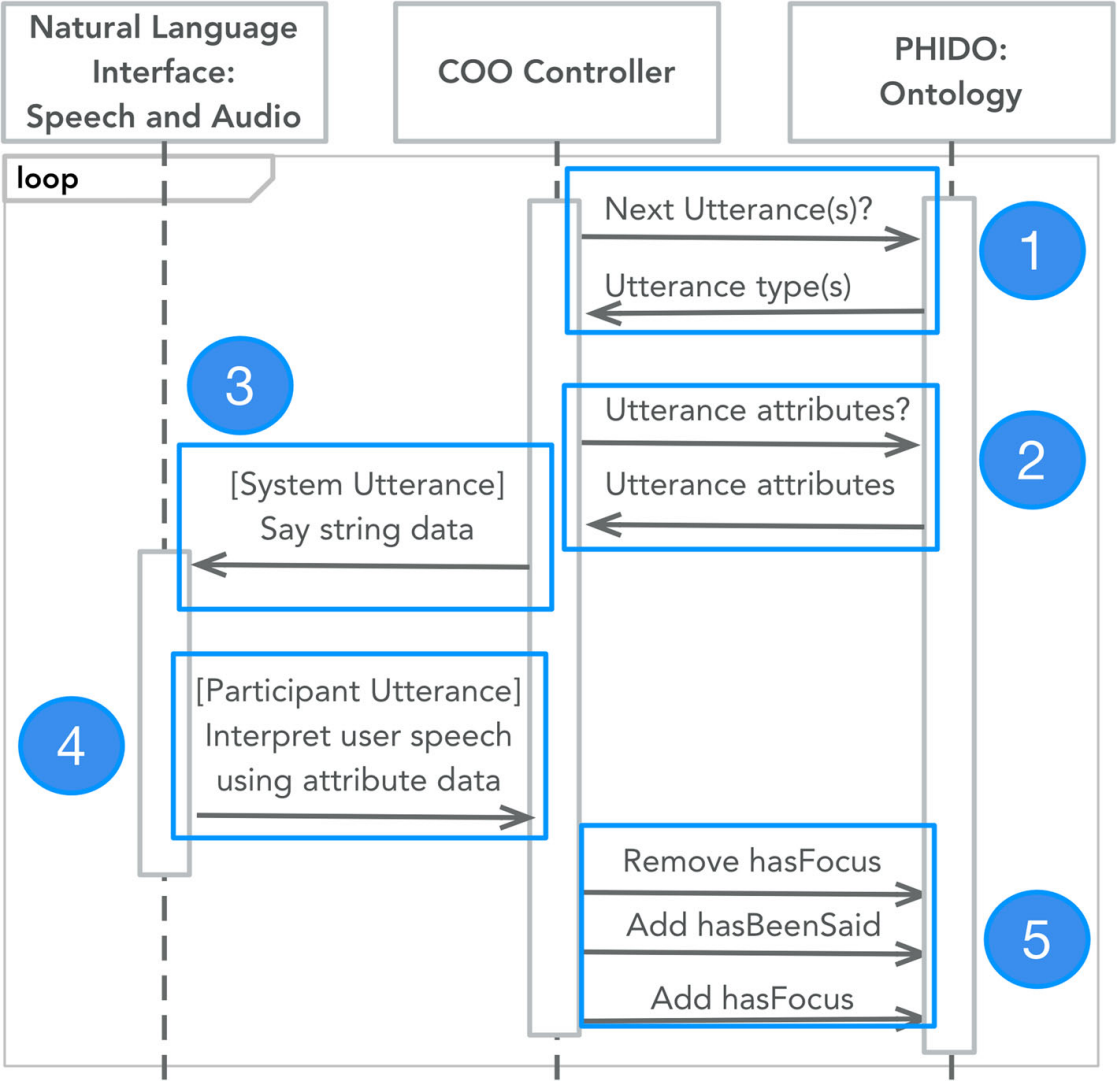
Macro-level summary of the dialogue interaction system of the COO engine. 1) The software controller queries for the next utterance based on the context of the dialogue, 2) and also query for next utterance data’s attribute information. 3) If the next utterance is a System Utterance the machine passes the utterance string data to the natural language interface. 4) If the next utterances are Participant Utterances, the natural language interface passes users’ utterance to determine what type of Participant Utterance. 5) The controller updates the context of the dialogue by updating the attribute data to progress the conversation

To summarize, COO implements a continuous loop where it first queries for the current position of the dialogue based on a data property (*hasFocus*). Afterwards, it queries for the next utterance instances and collects their data. If the utterance instance is an agent utterance (i.e., System Utterance, an utterance type evoked by a machine), the agent will communicate with the participant, or if it is a participant-related utterance (i.e., Participant Utterance, an utterance evoked by a human user of the agent) it will determine what type of utterance the user spoke (i.e., using the data associated with the utterance instance). Lastly, COO will update the position of the dialogue (*hasFocus*) and repeat. Figure 2 presents the macro-level implementation of the engine.

**Fig. 3:**
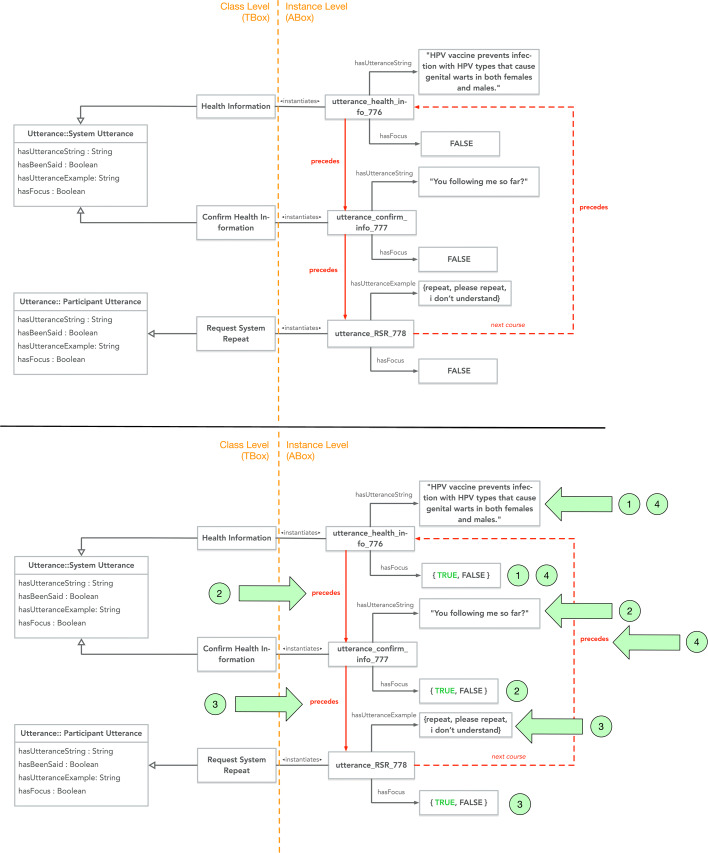
A brief example showing how PHIDO models and iterates a dialogue exchange for a user asking to repeat a piece of health information that was spoken by the system. Bottom level is an annotated version describing the dialogue flow. The red lines show the utterance data linked with *precedes* to coordinate the order of the utterance data. Numbers 1 through 4 shows the order of operations as utterance data traverses to the next utterance and flagging hasFocus to *true* to denote the placement of the conversation. Details are provided in *Dialogue Interaction Method* of the paper

To elaborate further on how PHIDO interacts with the system, Fig. 3 shows a narrow slice of the dialogue interaction model when a user expresses a desire to repeat the information that was said. On the left side of the figure is the class level (TBox) and right side is the instance level (ABox). One of the benefits of using an ontology-based method is we can utilize reasoning to determine what instance is being expressed in the dialogue interaction. In our system, we use the HermiT reasoner [17] to derive whether the utterance instance is a System or Participant Utterance. For each of the utterance instances there is a Boolean flag (*hasFocus*) to indicate to the machine where the discussion is placed. By default, this property for all of the instances is set to *false* unless it is the focal point of the dialogue. Figure 3 is annotated in green to show a walk-through of the process.

- **Step 1 of Fig. **3: The first utterance (*utterance_health_info_776*, a Health Information utterance) is set to *true*. Because this is a System Utterance, this is what the agent would declare to the user.
- **Step 2 of Fig. **3: The first utterance also has a property link (*precedes*) that leads to the next utterance (*utterance_health_info_777*, a Confirm Health Information utterance). This next utterance would be set to *true* and the previous is set to *false*. Similar in Step 1, this utterance is a System Utterance so the agent would ask the user “You following me so far?”.
- **Step 3 of Fig. **3: Again, the utterance has a property link leading to the next, and the system switches the *hasFocus* property for the next utterance instance (*utterance_RSR_778*, a Request System Utterance). This particular utterance is a System Utterance which has a property (*hasUtteranceExample*) for examples of what is expected to be said. The agent will use this to determine if the expected utterance from the user matches the examples. The agent’s dialogue engine uses string similarity and transcribed utterance from the speech interface to discern the type of Participant Utterance. For brevity, we only have one Participant Utterance instance in this example, but typically there would be branches of different expected Participant Utterances to which the agent could react. In one specific event, if a Participant Utterance type is a Question Utterance, the system will send the string data (the user’s question) to the question-answering subsystem (See Question-Answering Method) and wait for a response to continue.
- **Step 4 of Fig. **3: Same as the above steps, the *hasFocus* properties are updated, and the property link *precedes* leads to the next utterance. However, in this example, it returns to *utterance_health_info_776* since we are expressing the user’s desire to repeat health information that was evoked by the agent.

### Question-Answering method

**Fig. 4:**
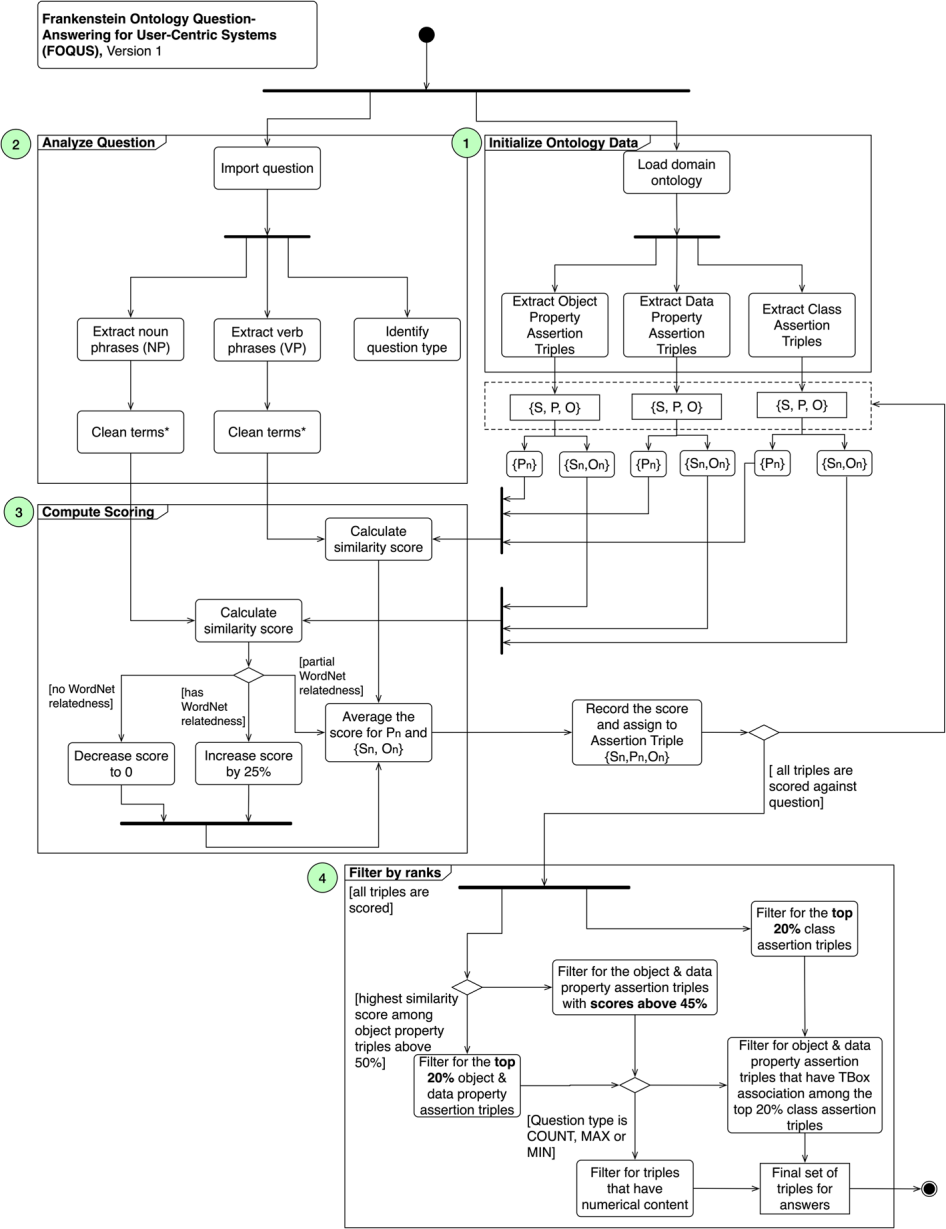
Process diagram outlining the implementation of the question answering system (FOQUS). 1) The knowledge base ontology is loaded by the QA system and assertion triples are extracted from the OWL file. This includes the domain and range of the assertion triples. 2) The question passed on from the COO is consumed by the QA system and is parsed by natural language processing methods for noun and verb phrases and denotes the question type. 3) The data from the question is compared to assertion triple data, and similarity scores are assigned to each triple. 4) The scored assertion triples are analyzed and filtered based on scoring rules. Details are provided in *Question-Answering Method* of the paper

In conjunction with COO, we developed a supporting question-answering sub-system to respond to questions by the user during a counseling session. Using a domain ontology, this QA subsystem called Frankenstein^2^ Ontology Question-Answering for User-centric Systems (FOQUS) queries an answer from a natural language question expressed by the user and transforms the resulting triples into a natural language form for the agent to communicate to the human user. The implementation is outlined and annotated in Fig. 4.

- **Step 1 of Fig. **4: FOQUS begins with importing an ontology knowledge base where Object Property Assertions, Data Property Assertions, and Class Assertion-based axioms are extracted. These axioms are generally the core domain knowledge to which user questions will query. Object Property Assertions are basic instance-level triples and Data Property Assertions are instance-level triples attributing data to the entity-level instances. Class Assertions are domain Tbox axioms. The delineation of these types of axioms would later serve in the ranking and selection of answers to be discussed in Step 4. After the specific axioms are extracted, the domain (i.e., subject), property (i.e., predicate), and range (i.e., object) are parsed and identified. This will later serve as tuples used for comparisons.
- **Step 2 of Fig. **4: FOQUS analyzes the user’s question by extracting the noun phrases and verb phrases and identifying the question type. The extractions of noun phrases and verb phrases are performed by Stanford Core NLP [18]. The question type identification is based on NLP-Reduce’s [19] classification which is rooted in looking at a series of keywords. For example, if the question contains “how many” or “the number of”, the question is classified as COUNT-based question. The classification has six categories - UNKNOWN, ALL (list all results), COUNT (count the results and give back the total), MAX (requesting maximum value), MIN (requesting the minimal value), and NUMERIC.
  In this step, FOQUS also cleans the terms from the noun and verb phrases. This would include removing special characters like underscores, removing duplicate words, removing common words (based on Oxford’s top 100 words), and normalizing each word to its root using MorphaStemmer [20].
- **Step 3 of Fig. **4: After extracting the axiom assertions from the ontology and the question data, FOQUS computes the similarity scores to determine what triples among axiom assertions are a probable answer for the question. Step 3 also describes the method for scoring. We utilized two methods for similarity: (i) vector-based approach using Numberbatch [21] as the vector model (cosine similarity), and (ii) string-based similarity. For the latter, we used the MongeElkan method [22, 23], which is the method that FREyA [24] uses for their similarity matching. By default, the Simmetrics library uses the Smith-Waterman-Gotoh for MongeElkan,^3^ instead of Jaro-Winkler, as its root metric.
  The process for determining similarity compares the predicate from a triple with the verb phrase from the question. Similarly, FOQUS uses entities (subject and object) from the triple and compares them with the noun phrases from the question. In certain cases, the verb phrase was non-existent in the question, so any comparison with the predicate of a triple would be ignored. All triples are sourced from the Object Assertions, Data Assertions, and Class Assertions.
  Initial experiments with a sample of questions indicated scoring using WordNet to enhance the resulting score. Using extJWNL [25], if two terms were deemed as synonymous within WordNet (using graph depth of 3), the score would be increased by 25%. If there are no synonym, hypernym, or hyponym between the terms, the score (even if there was some similarity indicated by the two methods), would be decreased to 0. Otherwise, the score would be left as is. Lastly, the average between predicate and entity scores is recorded for the axiom triple.
- **Step 4 of Fig. **4: The next step for FOQUS is filtering for the answer triple using the recorded scores. After all of the Object Property, Data Property, and Class Assertion triples are scored against the entities of the question, FOQUS captures the highest similarity score of the Object Property Assertion triple. If that top similarity score is above 50%, the top 20% of the Object Property and Data Property Assertions are captured. If this condition is not met, FOQUS defaults to filtering for the Object Property and Data Property Assertions above 45%. FOQUS utilizes the question type to determine additional filtering so if a question was identified as COUNT, MAX, or MIN, the system looks for triples among the selected Object Property and Data Property that have numerical content. For example, if the triple contained “one” or “1” in its label, that triple would be selected.
  If the question was not one of the aforementioned question types, FOQUS uses the top 20% scores of the Class Assertion triples for further selection. Using the URI for the triple’s domain, property, and range, FOQUS harnesses OWL-API and the reasoner (HermiT) to query for their respective TBox assertion. If that assertion was among the 20% of the Class Assertion triples, the Object or Data Assertion triple was selected. For example, the Object Assertion triple,*throat_cancer → affects → males*, is instantiated from *{Disease, Target} → {affects} → {Males, People of Gender, People}* (if we were to include the non-direct classes). If *Disease → affects → People* is among the top 20% from the Class Assertion triples, then *throat_cancer → affects → males* is selected.

The above method was developed using Java 8, using rdf4j [26], OWL-API [27], and HermiT reasoning [17] libraries. For QA, similarity methods employed string-based matching from SimMetrics [28] and vector-based comparisons using Numberbatch [21]. The implementation code was executed and tested within the Eclipse IDE’s console [29].

**Fig. 5:**
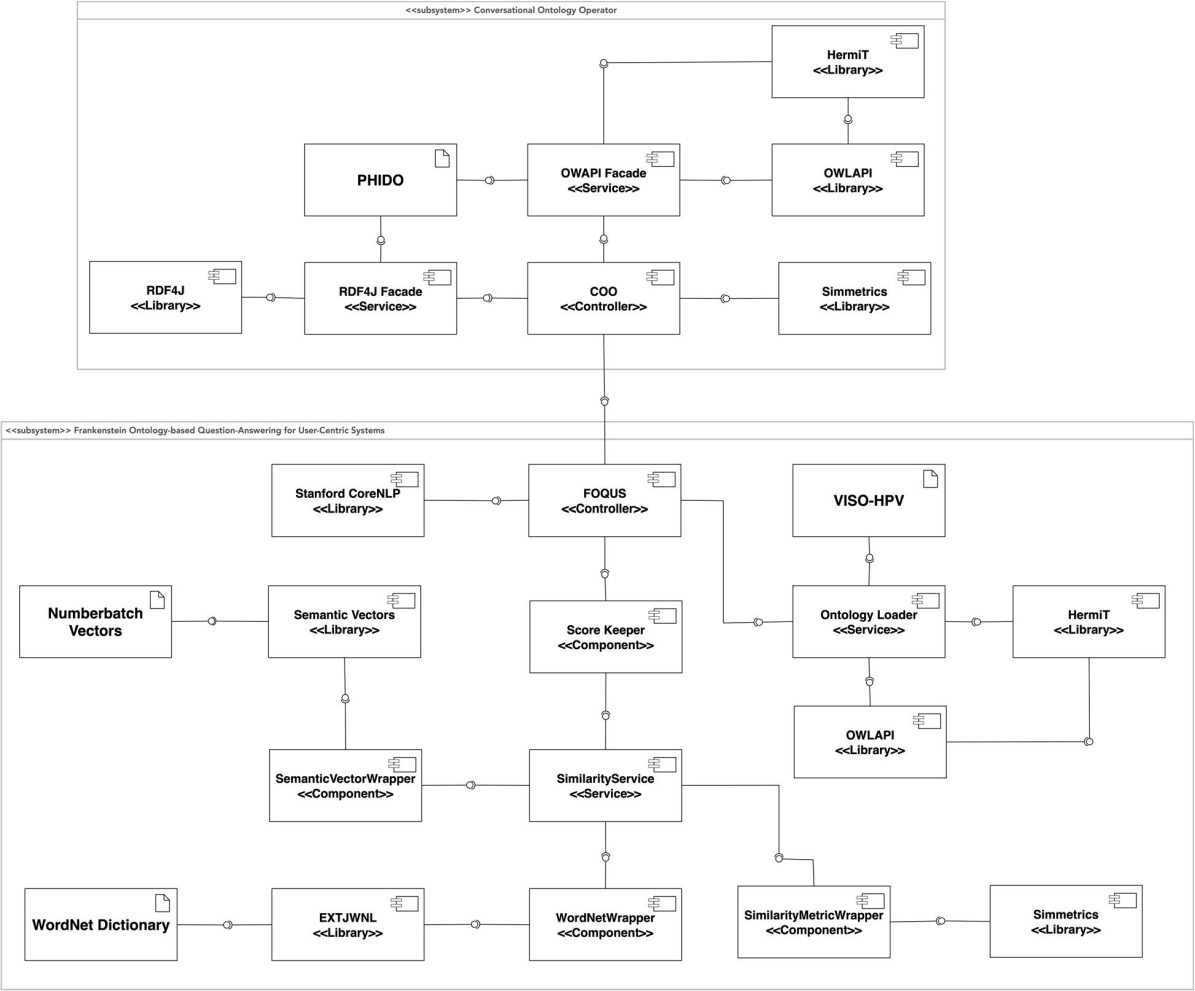
Component architecture outlining the various components in use for COO and FOQUS

Figure 5 shows the total component architecture of COO and FOQUS subsystem. As alluded to above, the controller of COO harnesses the PHIDO ontology using a combination of OWL-API, HermiT reasoner, and rdf4j to interact with the ontology. The COO controller operates the transition mechanism and also communicates with the FOQUS subsystem through its controller. The FOQUS controller primarily interfaces with the Score Keeper component, which handles the ranking of the assertion triples using similarity measure mechanisms - Numberbatch, WordNet, and Simmetrics. The FOQUS controller also interfaces with the VISO-HPV ontology for vaccine knowledge and the Stanford Core NLP library [18] for basic natural language processing functions (parts of speech tagging, chunking, etc.).

### COO functional evaluation

**Fig. 6:**
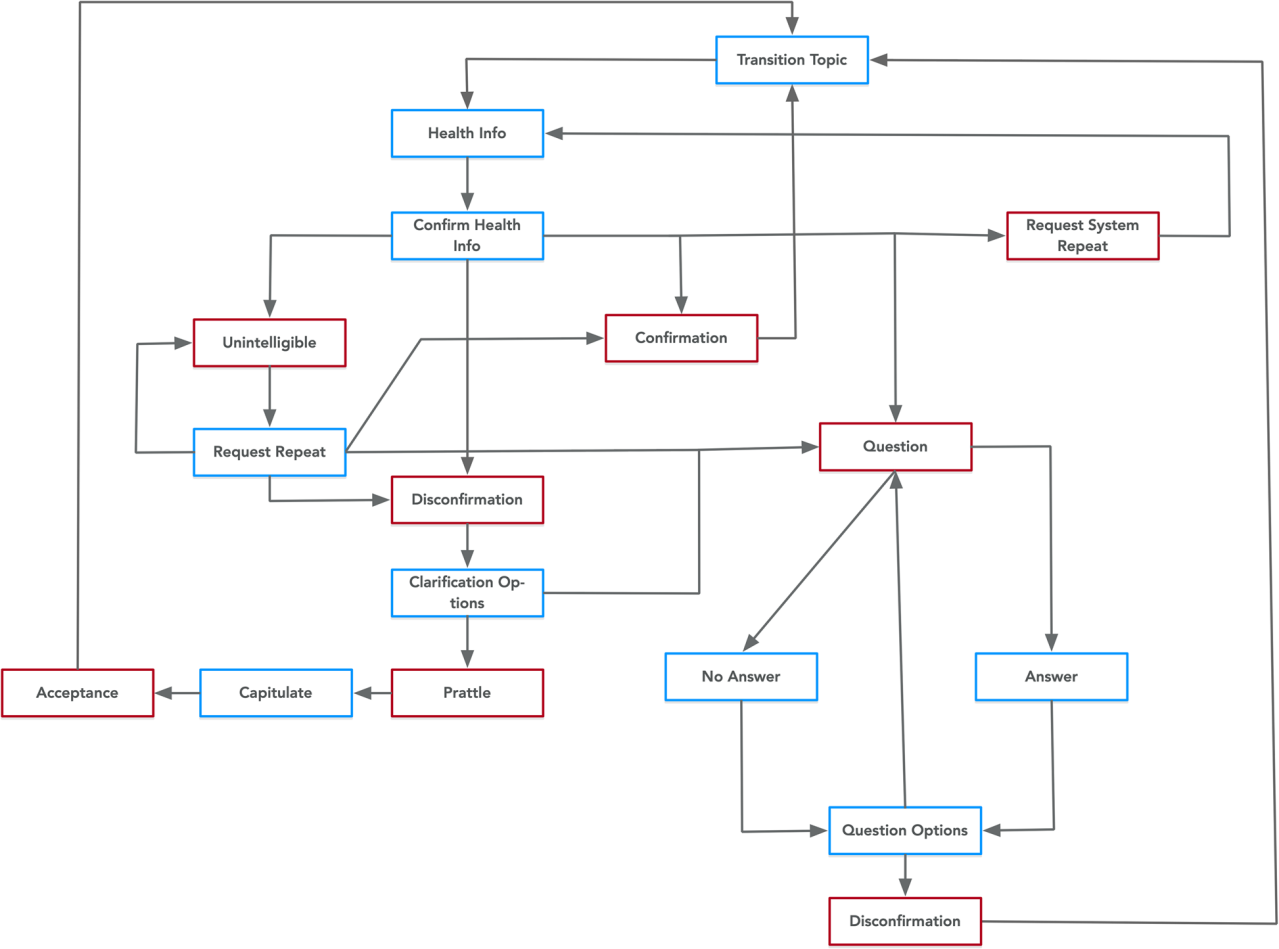
Health discussion interaction with blue squares indicating system utterances and red indicating participant utterances

Most of the dialogue interaction primarily involves communicating singular pieces of information about HPV and the HPV vaccine to the user. Figure 6 has a diagram that outlines the structure of this core dialogue exchange as our test example. To assess PHIDO’s ability to direct the COO engine’s interaction, we present the following questions:

Can the ontology-driven Conversational Ontology Operator:

1. *Impart a piece of health information (HPV vaccine-related) to the user?*
2. *Coordinate question-answering?*
3. *Transition the conversation to discuss a health topic*?

For the first objective, we tested several use cases. One of the use cases was to assess if the system can handle the user confirming they have heard the health information. Another use case was to review the system’s ability to manage if the user did not agree or did not understand the health information communicated to them. Other use cases included requesting to repeat the health information, switching to question-answering mode, and handling misunderstanding from the user. For the second objective, we tested the engine’s ability to provide the answer or no answer to the user’s question and also present options for the user to ask another question. The question answering for our test cases is always simulated, but in a later section we will discuss the automated question answering that we aim to integrate as a subsystem for COO. By default, all of the use cases end with transition to the next health topic to fulfill the third objective.

For testing purposes, we populated the PHIDO artifact with instances of sample utterances from our dialogue script used in a previous simulation study. In total, we had 19 instances of various Utterance classes. Each instance was linked using a specific object property *precedes*.

### FOQUS evaluation

To test FOQUS, we used participants’ questions from a simulated Wizard of OZ (WOZ) experiment [8]. These questions were unsolicited, and therefore, their authentic inquiries during the simulated counseling session. In total, we collected 53 questions that range from age appropriateness for the vaccine, gender-related questions, cost, etc. Some of the questions may have been mis-transcribed from speech recognition, but we kept it as is to imitate how the live system would process the question. Because of the possibility of mis-recognition of the utterances, FOQUS relies on the salient terms of the question (noun and verb phrases) to retrieve an answer. FOQUS provides two variants, one that employs vector similarity and the other string similarity matching. Both of these were tested against the 53 questions. Each of these questions was imported into the FOQUS system and answers were generated for each of the questions.

We enlisted the help of four evaluators (RL, DW, AZ, GX – young adults with premed or current medical student backgrounds) and asked them to qualitatively evaluate the question and answer pairs based on two criteria: the acceptability of the answer for the question (on a 5 point Likert scale) and whether the answer matches the question (2=yes, 1=partial, 0=no). The first criterion was devised to help us understand the presentation and composition of the question from triples. The second criterion helped us to determine if the system could answer the question with some degree of relevancy. We calculated Cohen Kappa’s inter-rater reliability [30] for both of these questions to determine the effectiveness of FOQUS.

## Results

### Conversational ontology operator results

**Fig. 7:**
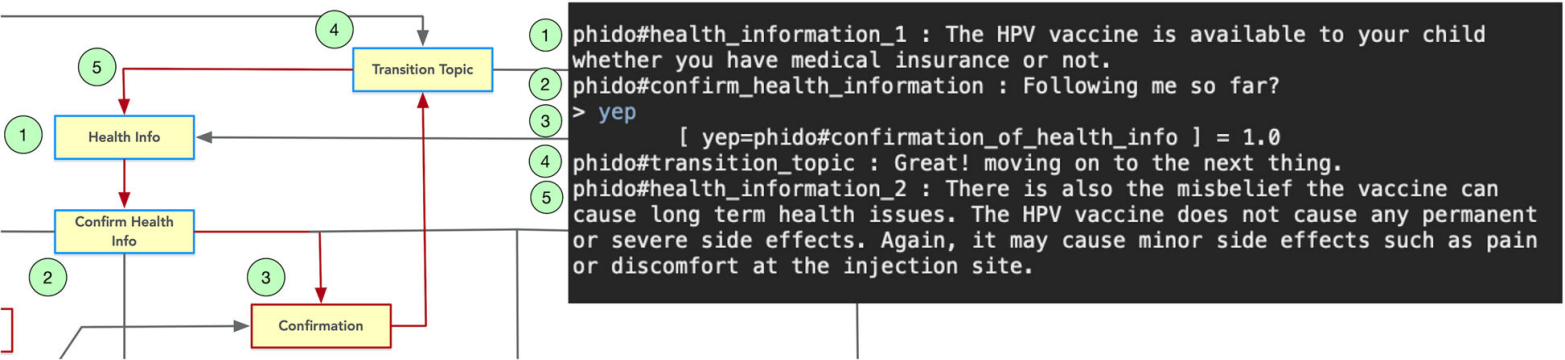
Dialogue interaction showing confirming health information. Red arrows indicate the path, and yellow box is the Utterance utilized in the result. See Fig. 6 for a complete view of the flow diagram

Figure 7 shows the text console demonstrating the test case revolving around the user indicating that he/she understands the health information communicated to them. For this case, the COO engine tells the user that the HPV vaccine is available irrespective of their insurance status and then follows up with the agent asking whether the user confirms this information. In this assessment, the simulated user responds with “yes” and the engine identifies it as Confirmation. The engine then continues to the next piece of health information in the dialogue.

**Fig. 8:**
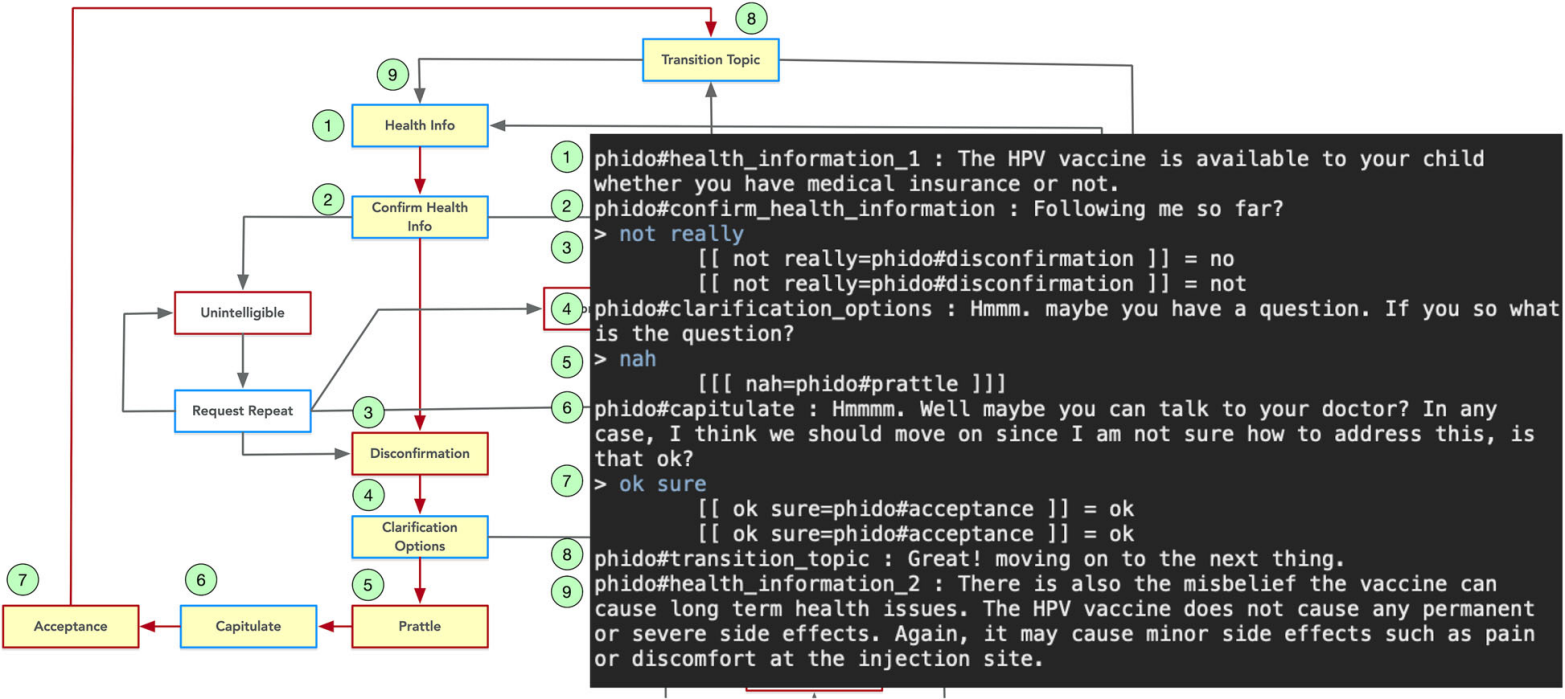
Dialogue interaction showing disconfirming health information. Red arrows indicate the path, and yellow box is the Utterance utilized in the result. See Fig. 6 for a complete view of the flow diagram

The contrast to the previous use case is if the user misunderstands or has some contentious notion of the information provided. Figure 8 outlines the test case with the simulated user saying “not really” in response to the health information uttered. The engine identifies the utterance as Disconfirmation and directs the agent to inquire if they have a question. The response is negative (e.g., “nah”), which the agent understands as prattle. The engine directs the agent to ask the user to move on to the next topic, saying that it is best to ask their health care provider if there is an issue.

**Fig. 9:**
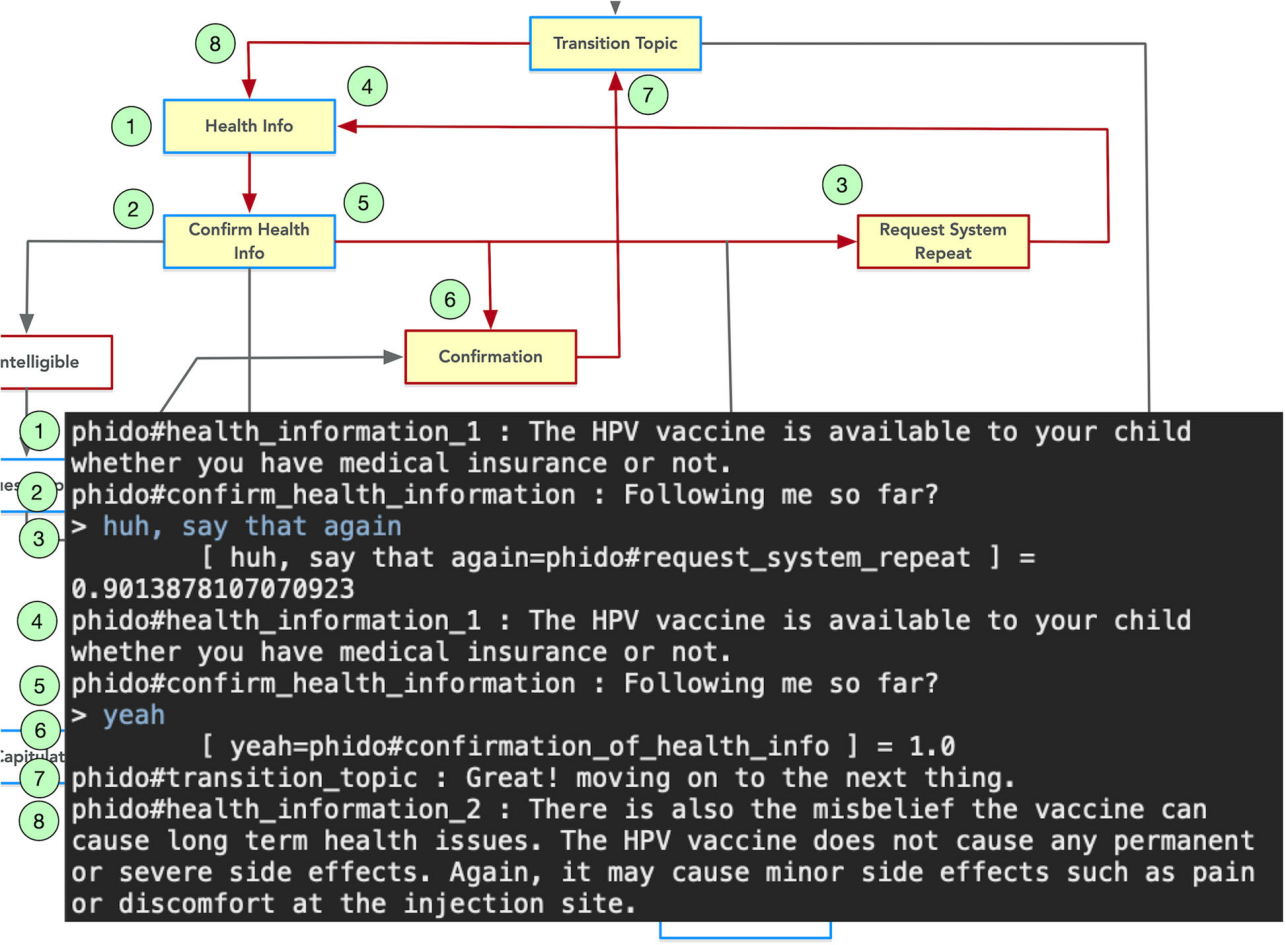
Dialogue interaction showing requesting the repeat of health information. Red arrows indicate the path, and yellow box is the Utterance utilized in the result. See Fig. 6 for a complete view of the flow diagram

If the user wants the agent to repeat the utterance, the engine can facilitate repeating the same health information (Fig. 9). In the test case, the agent repeats the same information after there is an utterance that is recognized as a request to repeat (Request_System_Repeat). The agent complies as instructed by the COO engine, and the test follows the course of the early use case (See Fig. 7).

**Fig. 10:**
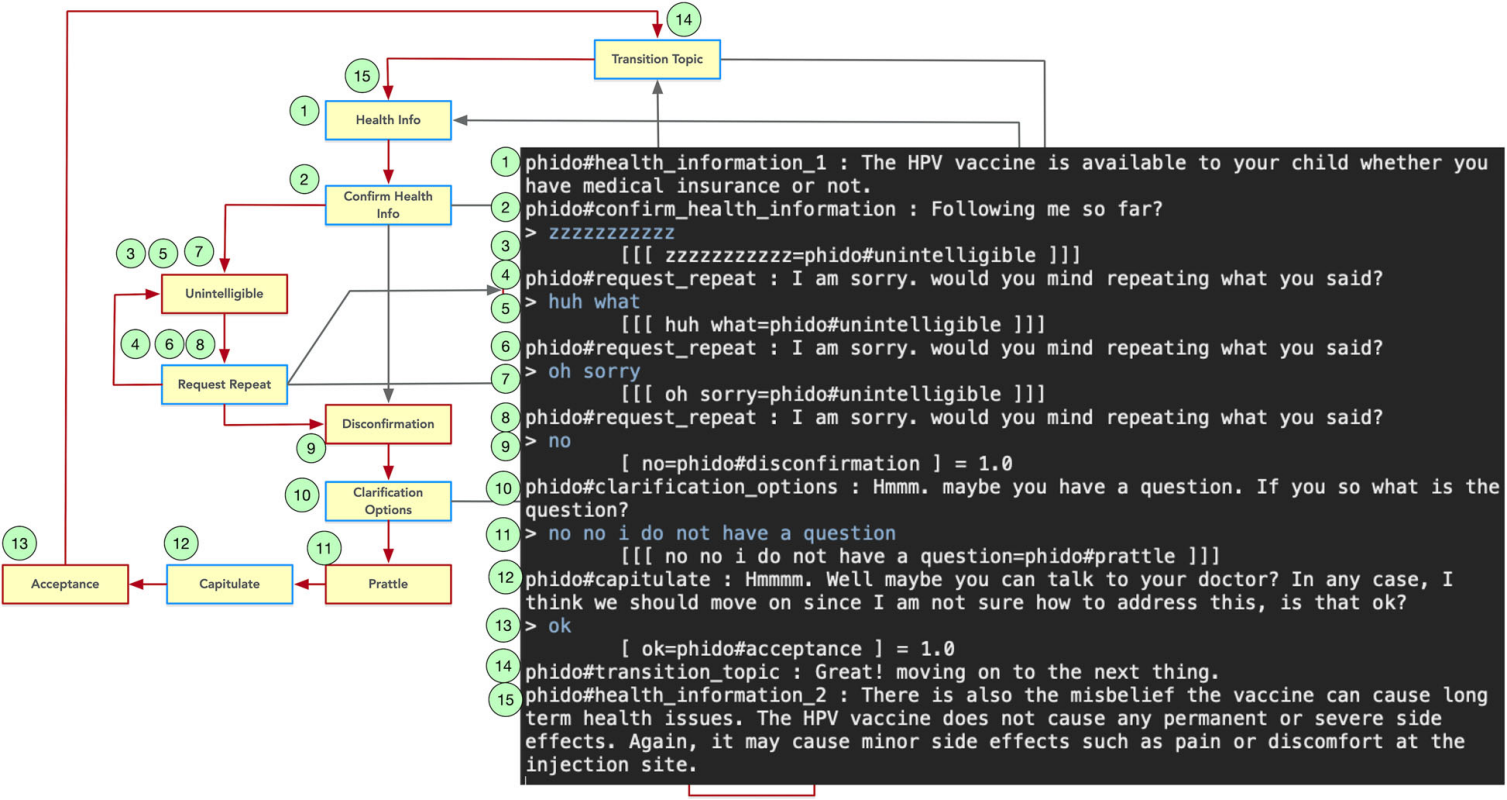
Dialogue interaction facilitating misunderstood utterances from the user. Red arrows indicate the path, and yellow box is the Utterance utilized in the result. See Fig. 6 for a complete view of the flow diagram

The COO engine, with direction from the PHIDO ontology, can handle situations where there may be a misunderstanding between the user and machine. Figure 10 shows an example, albeit a humorous situation, that highlights the engine’s ability to handle a use case where confusion may happen. Figure 10 has a series of exchanges from the user that are identified as the Unintelligible, which allows the agent to segue to the next health topic to discuss.

**Fig. 11:**
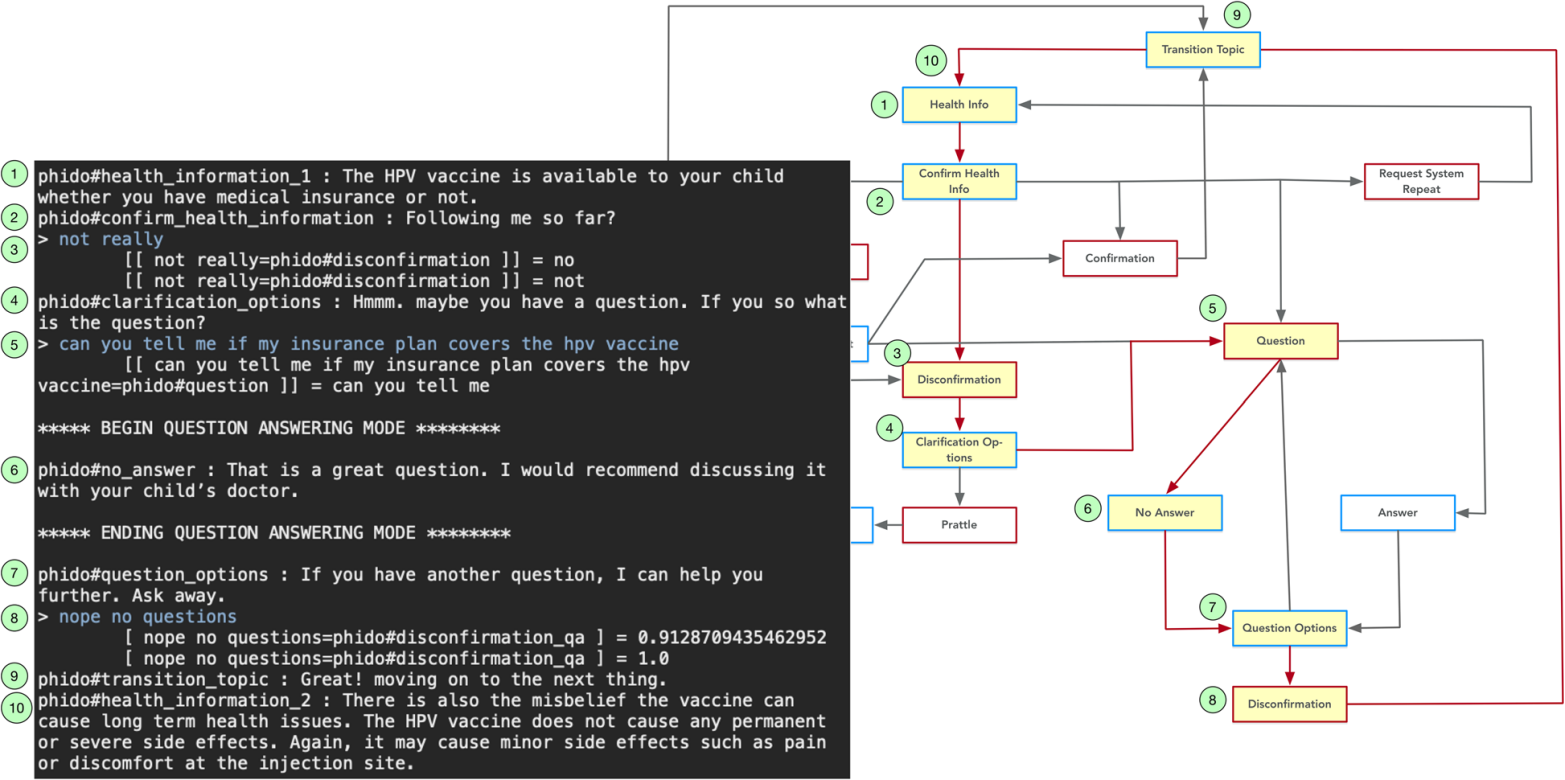
Dialogue interaction showing the transition from health information exchange to question answering mode (simulated). Red arrows indicate the path, and yellow box is the Utterance utilized in the result. See Fig. 6 for a complete view of the flow diagram

Figure 11 illustrates the test case for one of the ways the engine can switch to question-answering mode (to be facilitated by FOQUS). In this case, the user’s “not really” response is discerned as a Disconfirmation utterance type and the COO engine directs the agent to ask if the user has a question. The question is provided and successfully identified as a Question utterance type, which directs COO to switch to question-answering mode (simulated for test cases). The simulated question-answering system responds (the agent does not have an answer). The utterance “nope no question” is detected as a Disconfirmation utterance type, which signals the COO engine to continue. Figure 11 displays the details of the exchange for this use case.

**Fig. 12:**
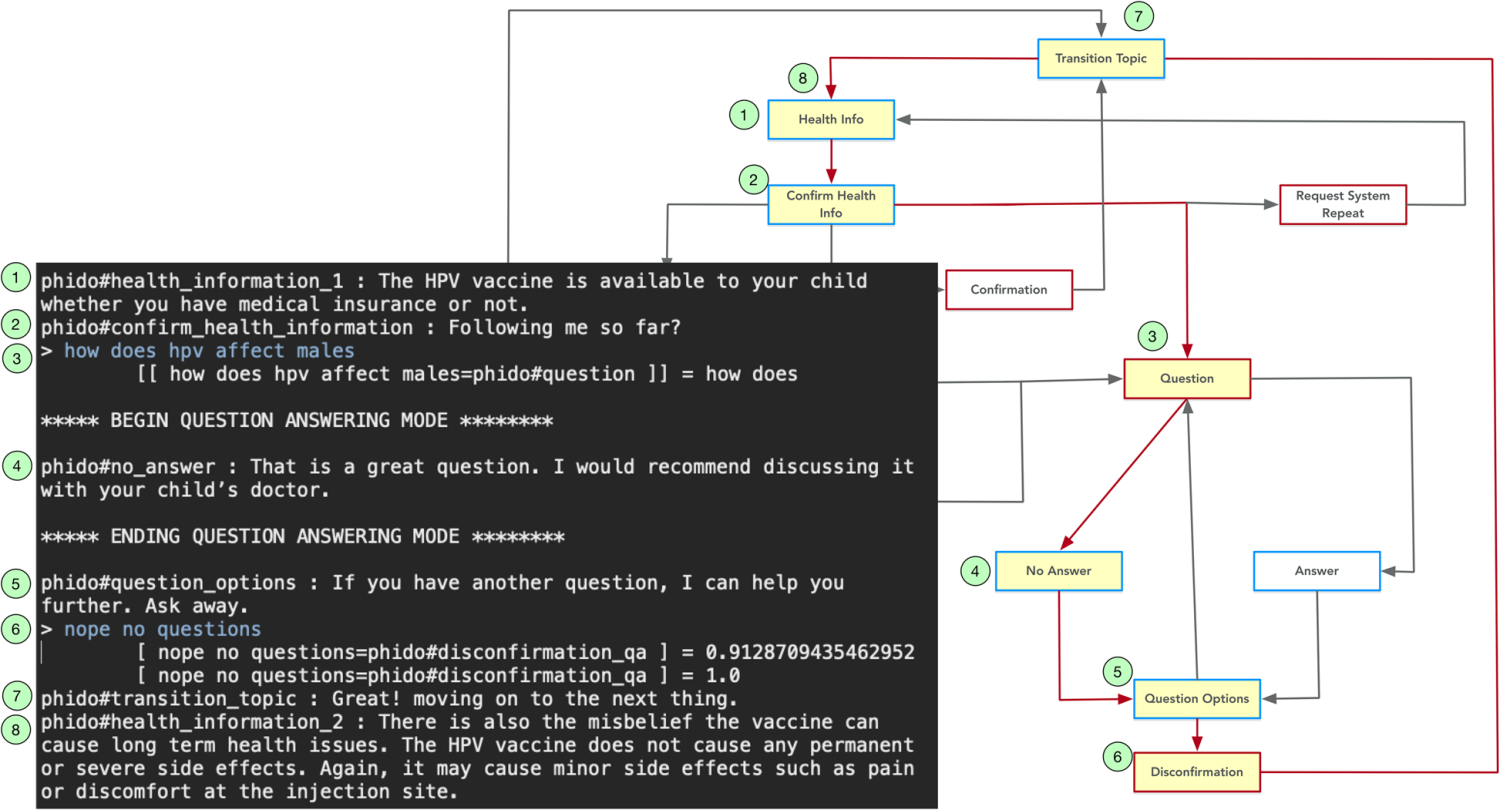
An alternate dialogue interaction showing the transition from health information exchange to question answering mode (simulated). Red arrows indicate the path, and yellow box is the Utterance utilized in the result. See Fig. 6 for a complete view of the flow diagram

Another way to direct the agent to question-answering mode in the dialogue interaction is demonstrated in Fig. 12. The use case is similar to the previous one, except the user asks a question when the agent inquires if the user confirms the information communicated to them.

**Fig. 13:**
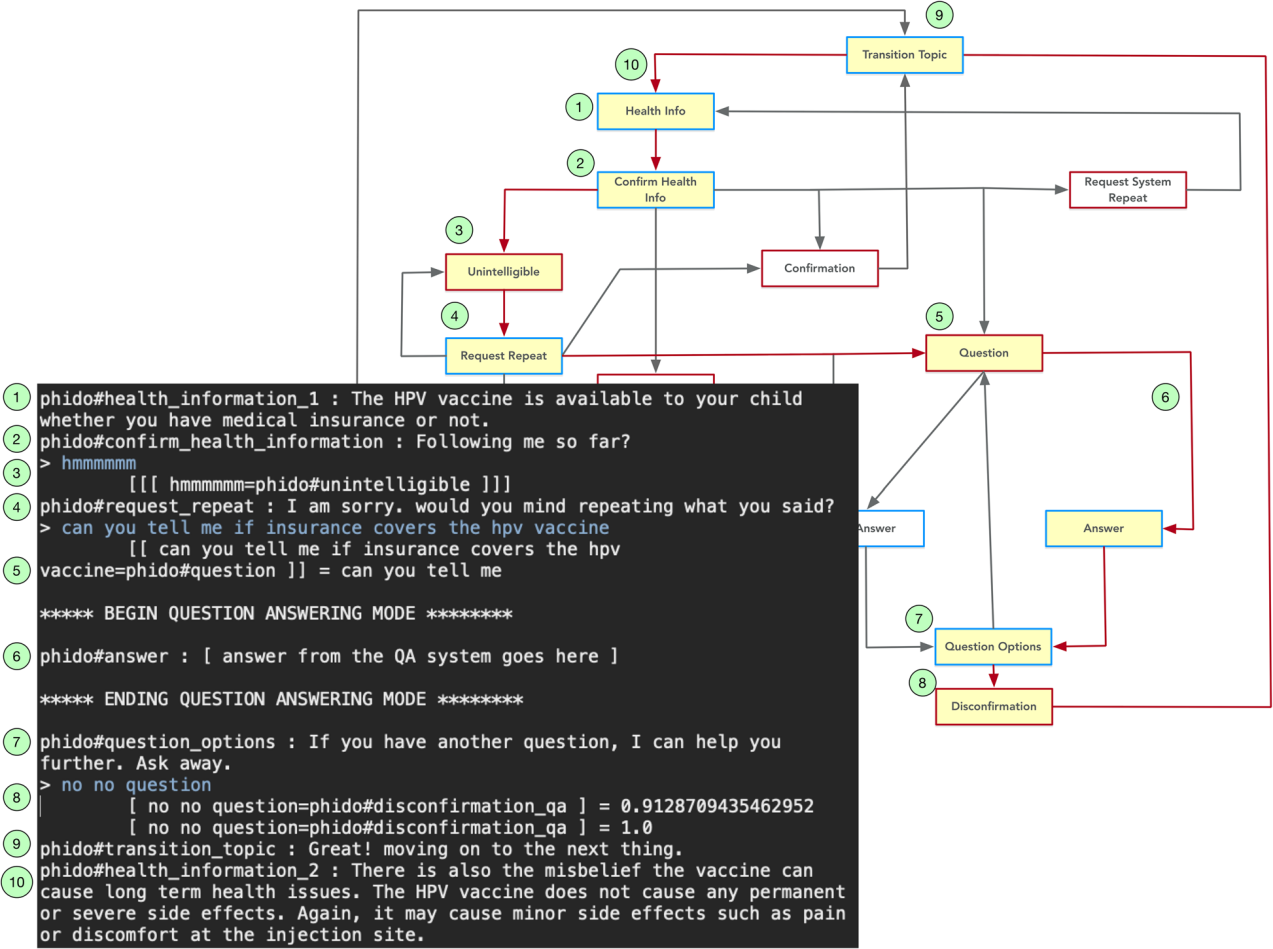
Dialogue interaction showing a question answered. Red arrows indicate the path, and yellow box is the Utterance utilized in the result. See Fig. 6 for a complete view of the flow diagram

**Fig. 14:**
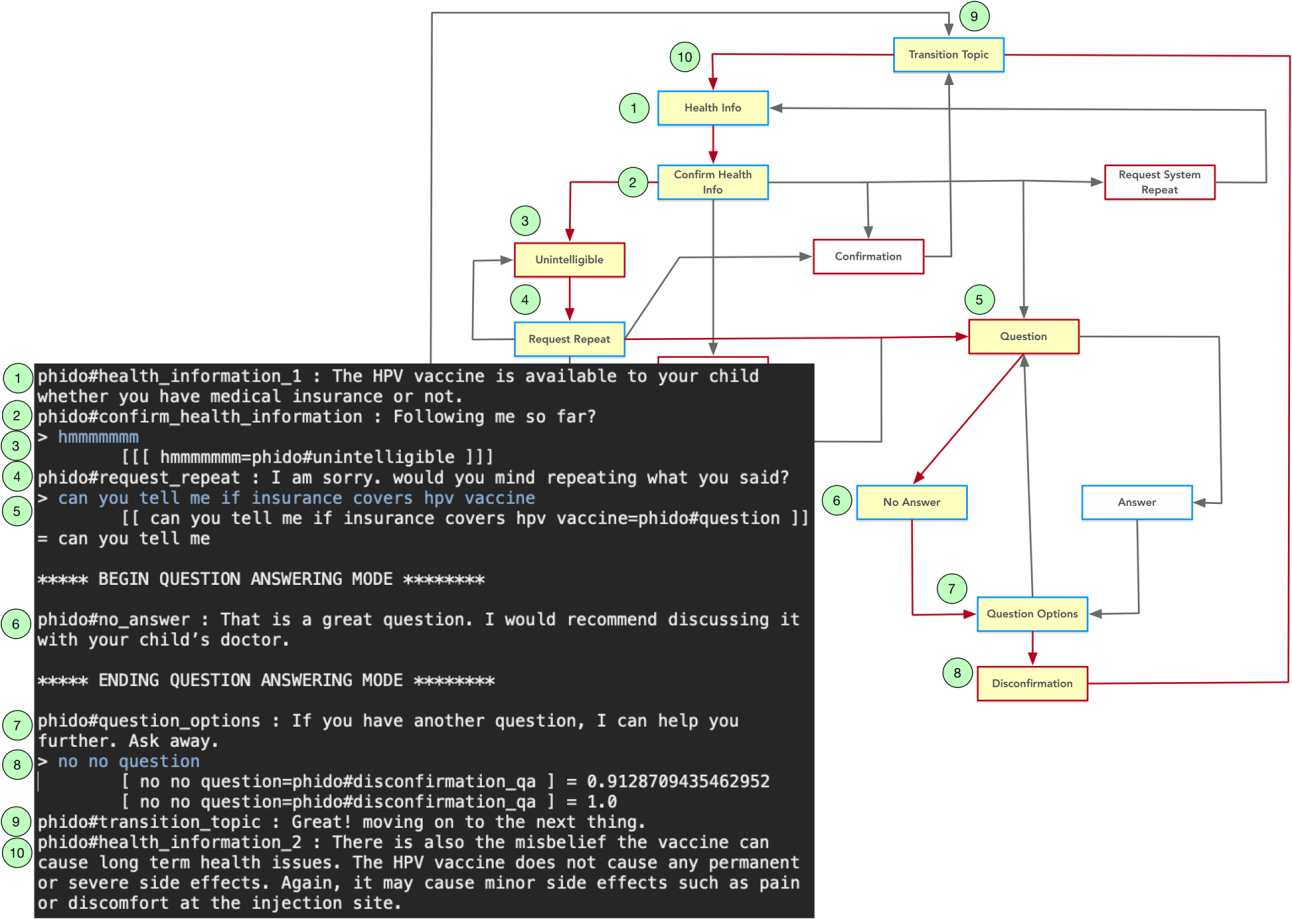
Dialogue interaction showing a question with no answer. Red arrows indicate the path, and yellow box is the Utterance utilized in the result. See Fig. 6 for a complete view of the flow diagram

Figures 13 and 14 show a similar dialogue interactions for answering a question, one in which the agent has a response to the question and the other in which the agent has no response to the question. The question regarding whether the HPV vaccine is covered by insurance (i.e., “can you tell me if insurance covers the hpv vaccine”) is recognized as a Question utterance type. This directs the system to switch to question-answering mode and the simulated question answering gives either an answer or no answer. Afterwards, the COO engine directs the agent to continue with the next piece of health information. Both Figs. 13 and 14 contain details of the exchanges for the use cases.

**Fig. 15:**
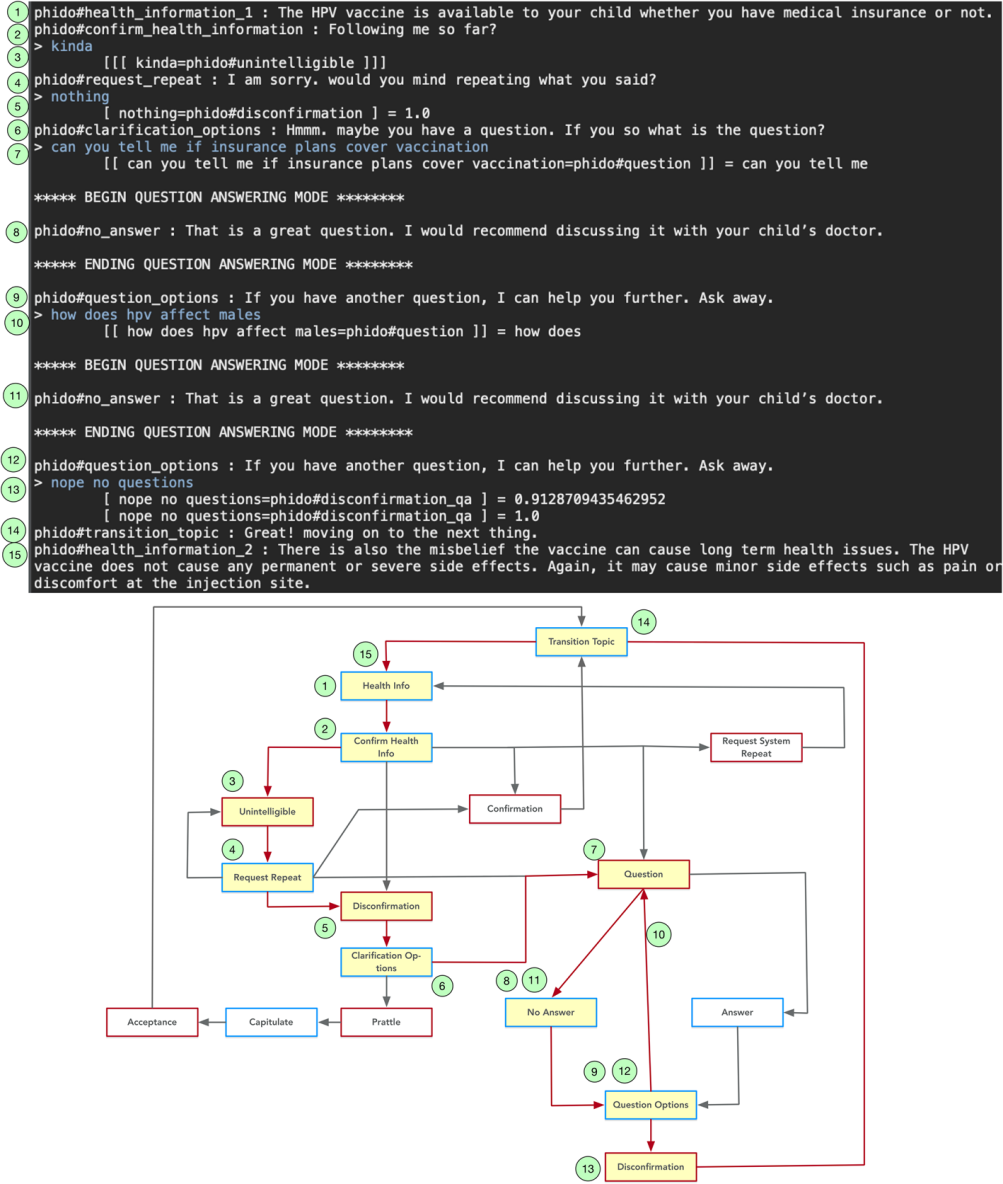
Dialogue interaction providing the user the option to ask another question. Red arrows indicate the path, and yellow box is the Utterance utilized in the result. See Fig. 6 for a complete view of the flow diagram

Within the question-answering interaction, COO can handle situations where the user may ask multiple questions. Figure 15 illustrates this use case starting from utterances that signal the COO engine to switch to the question-answering subsystem. The engine facilitates the interaction for the first question (“can you tell me if insurance plans cover vaccination”) and second question (“how does hpv affect males”), then segues to next health topic. Details of the sequence of the interaction are shown in Fig. 15.

In all of the above-mentioned use cases, by default, an instance of the next health information (*health_information_2*) is added to demonstrate COO’s movement from one Speech Task to another. In the examples provided, the agent transitions from one Discuss Health Task (for expressing that HPV vaccine is available regardless of insurance status) to another Discuss Health Task (for expressing there is a misbelief of long-term effects of the HPV vaccine).

### FOQUS results

We compiled the assessments for each of the questions from our evaluators. For the criterion regarding the acceptability of the answer, we normalized the ratings for degrees of acceptability (5 and 4) to 1, neutral (3) to 0, and degrees of unacceptability (2 and 1) to -1. For the criterion addressing whether the answer responded to the question correctly, ratings of answered (2) and partially answered (1) were recoded as 1 and ratings of unanswered (0) were coded as 0. In addition, we also tallied the non-normalized agreement (conservative) to further assess the performance of the question-answering system. Kappa’s inter-rater agreement was calculated on these recoded values among the four evaluators. In Table 1, we present agreement results for FOQUS.

**Table 1:** Agreement ratings for the question answering component

**FOQUS config**	**Acceptable answer**	**Perceived correctness**	**Perceived correctness**
		**(conservative)**	
vector variant	0.55	0.59	0.80
string variant	0.64	0.66	0.82

Table 2 presents the accuracy of FOQUS, along with the percentage of acceptability for the natural language composition of the answer. Similar to above, we calculated the accuracy of the question responses by coding the partially answered and completely answered ratings as 1, and unanswered ratings as 0. We also present the accuracy when coding completely answered as 1, and partially answered and unanswered as 0 (exact). Presentation of the answer was coded as 1 for degrees of acceptability, and as 0 for neutral and degrees of unacceptability.

**Table 2:** Accuracy of the question answering component

	**Vector variant**	**String variant**
Response answered the question		
*combined with partial*	0.72	0.70
*without partial (exact)*	0.54	0.50
Acceptable presentation of answer	0.50	0.49

For the acceptability of the answer, the semantic vector variant for FOQUS had a 0.55 agreement rating, while the string-based variant had a 0.64 agreement rating. For the perceived correctness of the answer, the vector-based variant had a 0.80 agreement rating, and the string-based configuration had a 0.82 agreement rating. The raw conservative agreement ratings were 0.59 and 0.66 for vector and sting variants, respectively.

FOQUS’ vector-based variant appears to perform slightly better for answer accuracy both in calculations of completely answered only (0.54 to 0.50) and calculations that include completely and partially answered (0.72 to 0.70). When considering the agreement from Table 1 where the string variant of FOQUS has slightly more agreement from evaluators, the better accuracy may not be conclusive. The same can be said for the presentation of the answer where the vector-based variant of FOQUS was slightly better than the string variant (0.50 to 0.49).

## Discussion

The Conversational Ontology Operator (COO) was supported through the use of PHIDO. By using PHIDO as the planning model for the dialogue engine, we were able to demonstrate the use of an ontology to control the flow of the dialogue and maintain the dialogue context at the same time. Three use cases were introduced - communicating one statement related to health information, facilitating the interaction for question answering, and transitioning to the next topic. In all of the use case tests, the engine was able to support the dialogue interactions. One important future goal for COO is to explore other consumer health domains like medication adherence counseling, behavioral health change, or mental health by simply constructing and importing new dialogue ontologies.

FOQUS provides question-answering abilities to answer sample questions from the simulation logs. It utilizes two variants (vector-based comparisons and string matching) to find matches of salient concepts of the question with the triples of the ontology. Irrespective of the configuration for FOQUS, the question-answering system did perform sufficiently in answering the questions from the chat logs collected from our Wizard of OZ experiment, with an accuracy ranging from 0.50 to 0.72 (depending on the variant or the inclusion of partially answered responses). With some promising initial results and a system foundation to build upon, refinement is needed to further improve FOQUS. We may explore natural language generation methods to better improve the transformation of triples to clear and natural answers. However, one limitation of this study is that we may need to factor in the impact of answers being uttered by a machine. For this, we need to assess FOQUS in a live environment with users and test its portability with other consumer ontology knowledge bases. Even though the HPV vaccine is now approved by the Food and Drug Administration for patients up to ages 45 [31], we also need to assess the answers by parents (decision makers for adolescents) to gain a more comprehensive assessment of FOQUS’ output.

The ultimate goal of our work is to utilize spoken dialogue systems to impact the uptake of the HPV vaccine, possibly leading to other positive consumer health changes beyond vaccine uptake. Several researchers have reported that health information technologies that employ behavior change theory are likely to be more effective in influencing users [32–36]. A review by Kennedy and colleagues [36], on health behavior change through interactive technology, mentioned the unique opportunity of ontology-based approaches to align with behavior change models, like the transtheoretical model [37] or motivational interviewing [38]. The reasoning capabilities of ontologies could provide an avenue for the personalization of consumer health. These aforementioned possibilities, like grounding in behavioral theory and tailoring, are some of the drivers for seeking an ontology-centric approach for this work. In our previous PHIDO study [16], the design of the ontology was influenced by our tested dialogue script [8] that was underpinned by the Health Belief Model which has a long history with vaccine uptake [39]. Our future goals are to further extend this software engine with ontologies that are related to user contextual information and health behavior change models that can link to the PHIDO in order to improve user experience with the conversational agent. Overall, we presume, since ontologies provides meaning behind the utterances for the machine, that the ontology-based approach has potential to do more sophisticated plan-based counseling and communication tasks.

## Conclusion

Our study introduces COO, an ontology-based software engine that employs the use of PHIDO from our previous study. We outlined some use cases that demonstrated the execution of the core conversational tasks by our software engine. Additionally, in support of the dialogue, we have also developed FOQUS, a question-answering subsystem for ontologies that uses our previously developed VISO-HPV, and demonstrated perceived ability to provide some sufficient responses to user questions from a Wizard of OZ experiment. Similar to our previous simulation studies, our next step is to test the software engine, coupled with a speech interface, on live participants in a clinical environment to examine its feasibility and usability.

## Data Availability

The datasets generated and/or analyzed during the current study are not publicly available due to stipulations agreed upon by the University of Texas Health Science Centers’ Committee for the Protection of Human Subjects but are available from the corresponding author on reasonable request.

## Abbreviations

PHIDO: Patient health information dialogue ontology
VISO-HPV: Vaccine information statement ontology for HPV
VISO: Vaccine information statement ontology
VIS: Vaccine information statement
COO: Conversational ontology operator
FOQUS: Frankenstein ontology question-answering for user-centric systems
HPV: Human papillomavirus
QA: Question-Answering
WOZ: Wizard of OZ

## Footnotes

1 The Wizard of OZ experiment entails a user interacting with a natural language interface through a software agent, but the software agent is remotely operated by a user giving the appearance of a live autonomous system. The purpose of this study is to collect early data and assess usability before development of an autonomous spoken software agent.

2 The inspiration behind the humorous name is due to its patchwork of methods and ideas from various classic QA for ontologies (NLP-Reduce, FREyA, PANTO, etc.).

3 https://github.com/Simmetrics/simmetrics/blob/59dc148f402da6a8a82ad8604a64fa35d1f70460/simmetrics-core/src/main/java/org/ simmetrics/metrics/StringMetrics.java
